# Improved Endurance Running Performance Following Haskap Berry (*Lonicera caerulea* L.) Ingestion

**DOI:** 10.3390/nu14040780

**Published:** 2022-02-13

**Authors:** Glyn Howatson, Gemma C. Snaith, Rachel Kimble, Gavin Cowper, Karen M. Keane

**Affiliations:** 1 Faculty of Health and Life Sciences, Northumbria University, Newcastle upon Tyne NE1 8ST, UK; g.snaith@northumbria.ac.uk (G.C.S.); gavin.w.cowper@northumbria.ac.uk (G.C.); karen.keane@gmit.ie (K.M.K.); 2 Water Research Group, School of Environmental Sciences and Development, Northwest University, Potchefstroom 2531, South Africa; 3 Population Health Sciences Institute, Newcastle University, Newcastle upon Tyne NE2 4HH, UK; rachel.kimble@newcastle.ac.uk; 4 School of Science and Computing, Galway-Mayo Institute of Technology, H91 T8NW Galway, Ireland

**Keywords:** human performance, anthocyanins, time to exhaustion, time trial, (poly)phenols, recovery

## Abstract

Background: Food high in (poly)phenolic compounds, such as anthocyanins, have the potential to improve exercise recovery and exercise performance. Haskap berries are rich in anthocyanins, but no research has examined the potential to improve human performance. The aim of this study was to determine the influence of Haskap berry on parameters of endurance running performance. Methods: Using a double-blind, placebo controlled, independent groups design, 30 male recreational runners (mean ± SD age, 33 ± 7 years; stature, 178.2 ± 7.2 cm; mass, 77.7 ± 10.6 kg; $\dot{V}$O_2peak_, 52.2 ± 6.6 mL/kg/min) volunteered to participate. Following familiarisation, volunteers visited the laboratory twice (separated by seven days) to assess submaximal, maximal and 5 km time trial running performance. After the first visit, volunteers were randomly assigned to consume either the Haskap berry intervention or an isocaloric placebo control. Results: There were modest changes in heart rate and $\dot{V}$O_2_ at submaximal intensities (*p* < 0.05). Time to exhaustion during the $\dot{V}$O_2peak_ test was longer in the Haskap group by 20 s (*p* = 0.031). Additionally, 5 km time trial performance was improved in the Haskap group by ~21 s (*p* = 0.016), which equated to a 0.25 km/h increase in mean running speed compared to the placebo control; this represented a >2% improvement in running performance. Conclusions: The application of this newly identified functional food to athletes has the capacity to improve endurance running performance.

## 1. Introduction

Plants have long since been utilised as medicinal sources and ergogenic aids [1,2]. As early as 668 BC the ancient Greeks reportedly used mushrooms, dried figs and various wine concoctions to enhance sporting performance [3]. More recently, there has been a growing research focus into bioactive fruit and vegetable compounds that might improve cardiovascular health [4] and physical performance [5,6] benefits. In particular, an emergent body of evidence suggests that dietary anthocyanins and (poly)phenols might improve physiological aspects of physical performance in recreational and well-trained athletic populations [7,8]. Anthocyanins are important pigments, often responsible for the red and blue colours in berries [9]. These non-nutritive compounds have been shown to exhibit antioxidant [10], anti-inflammatory [11], and vaso-modulatory actions [12], hence are thought to contribute, at least partly, to positively influencing performance following consumption of anthocyanin- and(poly)phenol-rich foods; e.g., New Zealand blackcurrant [13] and tart cherries [14,15,16,17]. 

Haskap (*Lonicera caerulea* L., commonly known as blue honeysuckle) is an emerging food that might also possess health-promoting properties due to the high anthocyanin and (poly)phenol content [18]. These deep-purple fruits have been consumed by the Ainu (indigenous people from Hokkaid Island, Japan) for centuries and are proposed to contribute to their life longevity [19,20]. Specifically, Haskap is a rich source of cyanidin-3-O-glucoside (C3G), which is a naturally occurring anthocyanin and often abundant in berries and cherries [21]. In vitro models of C3G and its metabolites have been shown to upregulate vascular endothelial nitric oxide synthase (eNOS) activity, which in turn improves endothelial function [22,23]. In support of this idea, a pilot study reported that Haskap berry (containing 400 mg of anthocyanins) reduced diastolic blood pressure and heart rate compared to a control [24]. In a rodent model, C3G supplementation increased indices of mitochondrial biogenesis and increased swimming to exhaustion performance [25]. In another murine model, C3G was shown to decrease inflammation in muscular dystrophy [26]. Furthermore, Rupasinghe et al. [18] reviewed evidence that showed Haskap berry and associated C3G reduce immune cell infiltration and the expression of the major pro-inflammatory cytokines such as interleukin-6 (IL-6), tumour necrosis factor-α (TNF-α) and prostaglandin E2 (PGE2), as well as a cyclooxygenase-2 (COX-2) enzyme macrophages. Finally, a recent study [27] has shown an upregulation of antioxidant gene and protein expression that were thought to be mediated by Nrf2 expression, and associated with preserved muscle function following strenuous resistance exercise. Collectively, the potential of C3G to affect vascular function, inflammation and oxidative stress make the (as yet untested) expectation tenable that aerobic performance could be improved in humans.

Given the aforementioned properties associated with C3G, it is plausible that Haskap berry could attenuate the development of exercise-induced oxidative stress and inflammation, as well as aid blood flow. This is likely to be more evident in activities where oxygen delivery is critical; consequently, it was hypothesised that Haskap berries would provide a performance benefit for endurance running. Hence, the aim of this proof-of-concept study was to determine the influence of Haskap berry on well-established and frequently used parameters of endurance running performance using a double-blind, placebo-controlled trial.

## 2. Materials and Methods

### 2.1. Participants

A total of 30 non-smoking males aged 18–45 years were recruited to take part in the study (mean ± SD age, stature and mass were 33 ± 7 years, 178.2 ± 7.2 cm, and 77.7 ± 10.6 kg, respectively). Inclusion criteria was determined by recreational runners who had completed a 5 km run in less than 25 min within the 6 weeks prior to the study. Exclusion criteria were allergies to fruit or dairy, currently taking any nutritional additional supplements (e.g., vitamins, antioxidant, protein drinks, creatine) or medication that might affect the study outcome and history of gastrointestinal, renal or cardiovascular disease. The study was conducted in accordance with the Declaration of Helsinki and ratified by the University’s Research Ethics Committee (HLS 26514) prior to participants providing written, informed consent. 

### 2.2. Study Design

This study employed a randomized, double-blind, placebo-controlled, independent groups design. An independent group design was used to reduce the risk of an extended wash-out period that could lead to changes in physiological variables underpinned by training status. Participants attended an environmentally controlled laboratory facility (accredited by the British Association of Sport and Exercise Sciences; BASES) on three separate occasions. To examine the influence of Haskap berry on aerobic performance, established performance tests (lactate threshold and $\dot{V}$O_2peak_) were used to ascertain selected determinants of endurance performance [27]. In addition, given the global success of weekly mass participation events like ParkRun [28] and its inclusion in the World Health Organisation’s (WHO) Action Plan on Physical Activity, we further employed a 5 km time trial to provide direct application for would-be end users. 

**Figure 1:**
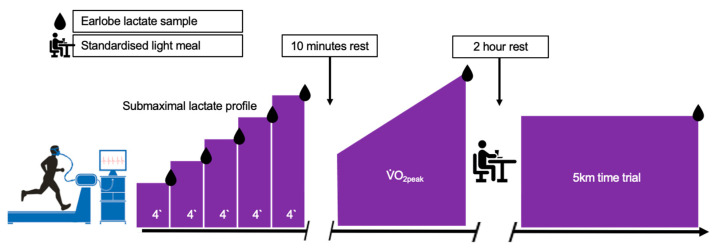
Schematic representation of the study design on Trial 1 and Trial 2.

On the first visit, volunteers completed a health and physical activity questionnaire to ascertain training status and check for any contraindications to participation. Following this, participants were familiarised with the treadmill (Pulsar, h/p/cosmos Sports & Medical GmbH, Germany) and completed a 5 km time trial (TT). Participants were then randomly assigned to either Haskap berry (HB) or an isocaloric placebo (PLA) group, 1:1 allocation. The second and third visits (Trial 1 and Trial 2) constituted the experimental trials, comprising a 5 km treadmill TT preceded by a submaximal lactate profile and maximal ($\dot{V}$O_2peak_) treadmill test (Figure 1). All exercise trials were performed at the same time of day between visits to avoid any influence of circadian variance and the environmental conditions during the visits were maintained at 19 ± 1 °C and 45–60% relative humidity. Participants completed a 24-h food and exercise diary prior to Trial 1 which was used to replicate their diet as closely as possible prior to Trial 2. Participants were also asked to arrive hydrated and to avoid strenuous exercise and alcohol consumption for 24 h and caffeine 12 for hours prior to both trials. The study was registered as a clinical trial with clinicaltrials.gov (NCT04837898).

### 2.3. Experimental Trials

During the experimental trials, resting blood pressure (BP) and heart rate (HR) were measured in triplicate using a validated [29], non-invasive, automated vital signs monitor (Carescape V100; General Electric, Chalfont St. Giles, UK) adhering to the guidelines specified by the European Society of Hypertension [30]. Participants completed the exercise protocol with a ~10-min break between the submaximal and $\dot{V}$O_2peak_ test and a 2-h break, in which volunteers were provide with a standardized light meal, before completing the 5 km TT. 

The submaximal test started at a speed approximating to ~2 km/h below the 5 km TT speed determined during familiarisation at a gradient of 1% to replicate the demands of outdoor running [31]. The speed was increased by 1 km/h every 4 min for ≥5 stages. The test was terminated when the blood lactate concentration reached the second inflection point or lactate turnpoint [32]. Following a ~10-min rest, participants completed a graded exercise test to determine $\dot{V}$O_2peak_. Participants ran at a speed approximating 2 km/h less than the individualised lactate turnpoint with a 1-min rolling start at a 1% gradient; the treadmill gradient was increased 1% every minute thereafter, until volitional exhaustion [33]. On completion, a 2-h rest period was allowed, during which volunteers were provided with a light meal (detailed below) and a further bolus of the Haskap berry or placebo control to ensure bioavailability was maintained. After the rest period and a short self-paced warmup, participants completed the 5 km TT at 1% gradient where speed was manually changed by the participant. Running speed and time were not visible during the TT, although feedback on distance covered was given at 1 km intervals.

### 2.4. Measurements

During the exercise protocol, capillary blood samples were collected from the earlobe at baseline, at the end of each 4-min stage of the submaximal test and immediately after the $\dot{V}$O_2peak_ test and 5 km TT. Samples were analysed immediately for blood lactate concentrations (Biosen C_Line, EKF Diagnostic, Barleben, Germany; CV < 1%). Heart rate was recorded continuously (H10, Polar, Finland) as was breath-by-breath pulmonary gas exchange (Vyntus CPX, Vyaire Medical INC, Basingstoke, UK) during all exercise tests. The $\dot{V}$O_2_ and HR were averaged over the last 30 s of each stage and in the case of maximal test, mean and peak HR and $\dot{V}$O_2_ were used to calculate HR and $\dot{V}$O_2peak_ parameters. Participants’ rating of perceived exertion (RPE) [34] was also assessed during each stage and at the end of each test.

### 2.5. Treatment and Dietary Control

Following the familiarisation visit, participants were randomly allocated to receive HB or PLA. The HB or PLA were mixed with a no fat yoghurt (100 g 0% Fat, Greek-Style Yogurt) to aid consumption [35,36]. Following Trial 1, participants took PL or HB mixed with yoghurt each morning for a total of 6 consecutive days before Trial 2. During Trial 2 participants were given their freeze-dried powder 1 h prior to commencing the submaximal test and an additional dose ~1 h before the commencement of the 5 km TT (during the 2 h break). This regimen was based on previous work that suggested highest bioavailability of (poly)phenols 1–2 h after consumption [37,38] having the potential to improve performance [5].

The HB intervention was a commercially available Haskap berry powder (Haskapa, Oxford, UK). The PLA was an unsweetened, artificially flavoured and coloured Black Cherry KoolAid (Kraft Foods, Chicago, IL, USA) with added maltodextrin to match carbohydrate and calorie content of the HB. According to independent analysis of the HB, the anthocyanin content was ~24.9 mg/g, (~150 mg/dose). The dose and duration are consistent with previous studies examining the effects of anthocyanin-rich foods on exercise performance [39]. To maintain blinding, participants were told the research was investigating the effects of a freeze-dried berry where 6 g of the food was pre-weighed and provided to participants in sealed sachets, along with enough yoghurt for the intervention period. Compliance was recorded by the return of each sachet and daily tick sheets. To assess blinding efficacy, participants were asked to guess the treatment they had received on trial completion. 

Throughout the study, participants were encouraged to maintain their habitual diet and exercise routines, however they were given verbal and written instructions to restrict (to a single portion a day) foods high in (poly)phenols and anthocyanins such as berries, red grapes and cherries (including extracts/juices), as well as red wine [40,41] for the study duration. At the experimental trials the participants were given a standardised light meal consisting of a sandwich and potato crisps (energy: 437 kcal; fat: 18.4 g; carbohydrate: 49.8 and protein: 16.5). The total amount of water consumed ad libitum during Trial 1 was noted and repeated on the subsequent visit.

### 2.6. Power Calculation and Statistical Analysis

Based on the smallest meaningful change ascertained from the intra-subject variability in a 5-km time trial (TT) of 20 s, with a typical error of 18 s [42] power of 80%, and α = 0.05, a total sample size of at least 14 per group would be required. Differences in group characteristics were determined using an independent samples t-test. To determine differences in submaximal, maximal and TT performance, an analysis of co-variance ANCOVA was employed to account for potential baseline differences between groups. An alpha of 0.05 was used as the significance level; effect sizes were interpreted as small, medium and large; 0.2, 0.5 and 0.8, respectively [43]. The 95% confidence intervals (95% CI) are also reported and all values are presented as means ± SD.

## 3. Results

Participants reported 100% compliance in both groups and no gastrointestinal issues. The volunteer characteristics are presented in Table 1 for the HB and PLA groups. There were no differences in baseline characteristics. A total of 15 participants completed the trial in each group. Two volunteers from the placebo did not complete the TT; whilst a further single volunteer from the Haskap group stopped early because they started at too fast-a-pace that could not be sustained. These participants are not included in the TT data analysis.

Macronutrient and total caloric intake did not differ between the first and second visits (1920 ± 577 kcal versus 1847 ± 656 kcal, respectively), and the macro-nutrient content was not different between groups or between trial (*p* > 0.05); macro- and micro-nutrient data are presented in Supplementary Material Tables S1 and S2. In the control group, nine participants did not know which treatment they were given, two guessed correctly and three thought they were on the intervention. In the Haskap group, seven did not know what intervention they were given, six guessed correctly and two guessed incorrectly. Based on these data, the intervention was well disguised in comparison to the placebo control.

### Submaximal Test

A summary of data is presented in Table 2 (lactate profile) and Table 3 (lactate turnpoint). There were small but significant (*p* < 0.05) reductions in HR at lactate threshold and lactate turnpoint in the Haskap compared to the placebo control group of 3 and 5 bpm, respectively. Furthermore, oxygen consumption was also lower (*p* < 0.05) at the lactate threshold (~2 mL/kg/min), but not at the lactate turnpoint, in the Haskap group compared to the placebo control.

**Figure 2:**
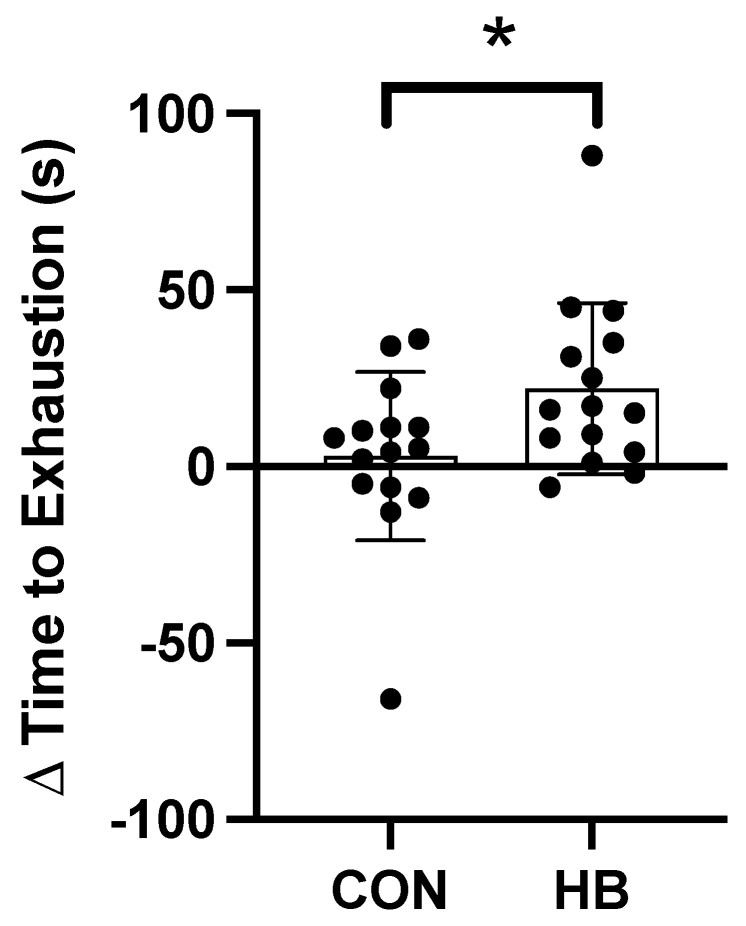
The change in time to exhaustion (TTE) during the $\dot{V}$O_2peak_ test in the placebo control (CON) and Haskap (HB) groups. Data are presented as mean ± SD; *n* = 30 (15 in each group); * denote significant differences between groups.

$\dot{V}$**O_2peak_ test**: A summary of data is presented in Table 4. There was an increase (*p* < 0.05) of 20 s in the time to exhaustion (TTE) during the $\dot{V}$O_2peak_ in the Haskap group compared to the placebo control (Figure 2). No other parameters were different between groups.

**Figure 3:**
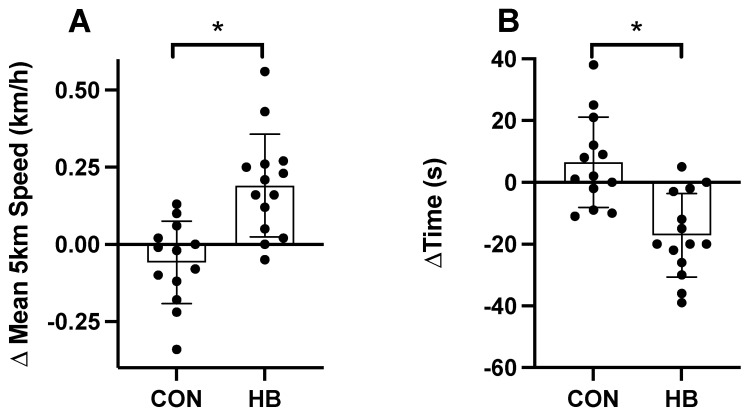
The change in time to mean speed during the 5 km time trial; TT (Panel **A**) and the change in 5 km TT performance (Panel **B**) in the placebo control (CON; *n* = 13) and Haskap (HB; *n* = 14) groups. Data are presented as mean ± SD; * denote significant differences between groups.

**5 km time trial**: A summary of data is presented in Table 5. There was an increase (*p* < 0.05) in mean speed (0.25 km/h) and a concomitant decrease (*p* < 0.05) in the 5 km time of 20.9 s in the Haskap group in comparison to the placebo control (Figure 3). No other parameters were different between groups.

## 4. Discussion

The aim of this study was to investigate the effects of Haskap berry on endurance running performance parameters. It was hypothesized that Haskap berry would improve parameters associated with aerobic running performance. The results showed lower HR and $\dot{V}$O_2_ at lower intensity exercise (lactate threshold), but importantly, there was a discernible improvement of ~2.2% in time to exhaustion running performance with acute Haskap consumption during the $\dot{V}$O_2peak_ test (~20 s). These small effects were mirrored by an improved 5 km time trial performance of ~21 s (equating to 0.25 km/h in mean running velocity), which represents a meaningful change in the context of human running performance.

Previous investigations have consistently demonstrated positive effects of anthocyanin and (poly)phenol-rich foods on indices of oxidative stress, inflammation and muscle recovery [5,15,16,17]. A recent systematic review with meta-analysis demonstrated anti-oxidative, anti-inflammatory and functional recovery properties [44] following consumption of anthocyanin-rich foods; however, the data on exercise performance is relatively small and far less clear (Myburgh, 2014). Importantly to this study, a systematic review with meta-analysis [45], showed that the use of phenolics for a minimum of seven days increased exercise performance by 1.90% (95% CI 0.40–3.39), which is in close agreement to the performance improvements (2.2%) seen with Haskap berry when compared to a placebo control in the time to exhaustion during the $\dot{V}$O_2peak_ test. Similarly, Cook, Myers [13] examined the effects of a seven-day New Zealand blackcurrant (NZBK) extract supplementation (105 mg anthocyanin·day^−^^1^), on 14 trained cyclists’ performance (16.1 km time-trial). In close agreement to the aforementioned meta-analysis [45], cyclists showed a 2.4% improvement with blackcurrants [13], and 2.7% with beetroot [46] in 16.1 km cycling time trial performance. Additionally, Murphy, Cook [47] reported a performance increase of 0.82% with NZBK following two, 4 km cycling time-trials separated by 10 min. Collectively, these data are promising, but the latter study arguably lacked specificity to a sporting context because there are no known competition scenarios of this nature. 

In relation to running performance, seven-day NZBK supplementation showed a 10.6% increase (total distance) during a treadmill running to exhaustion [48], although the translation of these large changes should be treated cautiously given the intermittent sprint nature of the protocol and the scope for variability might be high. The same research group later investigated the same intervention on the Loughborough Intermittent Shuttle run test and showed no change, except for a preservation of sprint speed in the latter parts of the test [49], which could be interpreted as greater fatigue resistance. The current study showed some similarities but used established aerobic performance measures and showed improved time to exhaustion and improved 5 km time trial performance, which could be reflective of the previous observations on the preservation of sprint ability. Importantly, this study used activities that many runners will conduct and hence has direct application to the wider community. 

There is a paucity of data that examines the influence of phenolic-rich compounds on human exercise performance; rather there is far more work in exercise recovery, cognition and vascular function. Consequently, the application for (poly)phenols on human performance is an exciting new area for exploration. The current data provide some optimism for Haskap berry and other (poly)phenolic-rich fruits to exert performance benefits for humans in aerobic exercise, but further work should confirm these data and explore the potential application for anaerobic or resistance exercise paradigms.

Providing a mechanistic underpinning for these observations is not straightforward, however previous work has suggested that anthocyanin-rich foods increase fat oxidation [13], which might have glycogen sparing properties for work done in the later parts of high intensity exercise. However, given that HR and $\dot{V}$O_2_ were lower at the moderate exercise intensity (lactate threshold) it seems unlikely that fat was a preferential fuel source because fat oxidation has a greater O_2_ cost than carbohydrate. These observations were not consistent at higher intensities (lactate turnpoint), which concur with previous reports following tart cherry supplementation [50]. Given that Haskap contains C3G, which was shown to increase mitochondrial biogenesis pathways, improve muscle function and increase exercise performance in rodents [25,26], and that Haskap berry has directly shown to improve vascular function [24], it is feasible that performance was improved via C3G mediated pathways modulating endothelial function [22,23]. We therefore speculate that better vascular function leads to a more efficient use of O_2_ at lower intensities, leading to a greater preservation of W prime for work completed in higher intensity domains above critical power, which could explain the improved time to exhaustion and time trial performance; this idea should be explored more fully and systematically in the future.

One other plausible explanation is the antioxidant properties provided by (poly)phenols that reside in Haskap berries. These (poly)phenols are proposed to reduce fatigue and increase exercise performance [51,52]. A recent addition to the literature demonstrated reduced in vitro intracellular free radicals of fibroblasts with exposure to isolated Haskap berry extracts [53], but this has yet to be demonstrated in humans. Nonetheless, there is now convincing evidence that these types of phytochemicals induce enzymatic antioxidants, such as superoxide dismutase, by activation the transcription factor nuclear factor-erythroid-2-related factor 2; Nrf2 [54,55] that enable defence against redox challenges. Although data are limited in humans, a recent investigation [56] in an exercise paradigm showed that tart cherries (high in C3G) upregulated antioxidant gene and protein expression that were thought to be mediated by Nrf2 expression, and preserved muscle function following strenuous exercise. Lastly, a reductionist approach to elucidate a single mechanism might not be pragmatic and future research should attempt to look at various integrated facets of the proposed mechanisms in order to gain a richer picture of the impact of dietary anthocyanins on exercise performance and recovery.

Although the study design was well controlled, there are limitations that should be acknowledged. Firstly, the bioavailability of phenolics afforded by the Haskap berry was not ascertained, so it is not possible to determine the plasma anthocyanins and phenolic acids available prior to the exercise challenge. However, previous work using anthocyanin-rich cherries showed plasma bioavailability to peak around 1–2 h post consumption [37]. Notwithstanding, it would be helpful for future work to ascertain the optimal dose to provide a beneficial effect on exercise performance. In addition, the lack of inflammatory and oxidative stress indices further limits the mechanistic insight of the positive effect seen in running performance. Future work should use the current research as a platform to elucidate the potential mechanisms underpinning changes in human performance. The study design allowed participants to be free living but restrict anthocyanin-rich foods to a single portion per day; however, importantly, volunteers did maintain a similar diet and hydration status (as best as possible) for the time preceding both laboratory visits. This control measure might have restricted habitual (poly)phenol intake in some volunteers; however, the self-reported portions of fruit and vegetables and total caloric value was not different between groups or between visits. Equally, we did not empirically establish hydration status beyond self-reporting, so it is possible hydration status was not equitable between visits. As previously mentioned, an array of biomarkers designed to gain mechanistic insight would be advantageous in future research, but this study does provide a basis to inform future investigations.

## 5. Conclusions

The current study showed for the first time that Haskap berry consumption can improve time to exhaustion and 5 km time trial running performance by >2%, compared to a placebo control. Modest changes at lower intensities suggest better exercise efficiency that might be made possible through improved vascular function or management of exercise-induced oxidative stress, although these remain to be demonstrated. These data on Haskap berry add to the growing body of evidence that dietary (poly)phenolic-rich foods could be helpful to enhance athletic performance, and critically offer exercisers a practical, non-pharmacological, food-based solution to support training and competition.

## Figures and Tables

**Table 1:** Participant characteristics for the Haskap and Control groups.

	Age (years)	Stature (cm)	Mass (kg)	5 km TT (s)	Training Volume (min/week)
**Haskap (*n* = 15)**	30 ± 8	176.5 ± 5.3	75.0 ± 10.9	1377 ± 192	281 ± 142
**Control (*n* =15)**	35 ± 6	179.8 ± 8.6	80.4 ± 10.0	1299 ± 141	245 ± 156

**Table 2:** Data from the submaximal test for lactate threshold (LT) parameters before and after the intervention of Haskap or placebo control.

			ANCOVA Adjusted for Baseline			
	Control	Haskap	Difference (95% CI)	F	*p*-Value	Effect Size η^2^
**Speed @LT (km/h)**						
Pre	11.7 ±1.9	12.1 ± 1.8	0.26 (−0.68–0.22)	1.062	0.312	0.038
Post	11.7 ± 1.9	12.4 ± 1.7				
HR @LT (bpm) *						
Pre	158 ± 10	151 ± 10	3.3 (0.67–5.98)	6.639	0.016	0.197
Post	159 ± 10	149 ± 9				
**RPE @LT**						
Pre	11.6 ± 2.1	12.1 ± 2.3	0.03 (−0.94–0.88)	0.005	0.944	<0.001
Post	11.4 ± 2.1	11.8 ± 2.0				
Relative $\dot{V}$O_2_ @LT (mL/kg/min) *						
Pre	39.6 ± 5.7	40.7 ± 5.0	2.2 (0.67–3.69)	8.799	0.006	0.246
Post	40.4 ± 5.6	39.3 ± 5.3				
Absolute $\dot{V}$O_2_ @LT (mL) *						
Pre	2946 ± 468	3248 ± 413	131 (16.6–246)	5.517	0.026	0.170
Post	2998 ± 404	3139 ± 440				

Data are presented as mean ± SD; *n* = 30 (15 in each group). * denotes significant differences between groups.

**Table 3:** Data from the submaximal test for lactate turnpoint (LTP) parameters before and after the intervention of Haskap or placebo control.

			ANCOVA Adjusted for Baseline			
	Control	Haskap	Difference (95% CI)	F	*p*-Value	Effect Size η^2^
**Speed @LTP (km/h)**						
Pre	13.3 ± 1.7	14.1 ± 1.6	0.177 (−0.20–0.56)	0.925	0.345	0.033
Post	13.5 ±1.7	14.0 ± 1.7				
HR @LTP (bpm) *						
Pre	171 ± 8	169 ± 7	5.3 (2.82–7.69)	19.534	<0.001	0.420
Post	172 ± 8	165 ± 6				
**RPE @LTP**						
Pre	14.9 ± 1.5	14.7 ± 2.2	0.14 (−0.49–0.76)	0.201	0.657	0.007
Post	15.0 ± 1.2	14.7 ± 2.1				
**Relative $\dot{V}$O_2_ @LTP (mL/kg/min)**						
Pre	44.6 ± 6.1	46.0 ± 5.0	0.6 (−0.84–2.11)	0.786	0.383	0.028
Post	45.2 ± 6.3	45.8 ± 5.1				
**Absolute $\dot{V}$O_2_ @LTP (mL)**						
Pre	3328 ± 522	3676 ± 403	0.45 (−112.10–113.01)	<0.001	0.993	<0.001
Post	3358 ± 478	3656 ± 378				

Data are presented as mean ± SD; *n* = 30 (15 in each group); * denote significant differences between groups.

**Table 4:** Data from the $\dot{V}$ O_2peak_ test before and after the intervention of Haskap.

			ANCOVA Adjusted for Baseline			
	Control	Haskap	Difference (95% CI)	F	*p*-Value	Effect Size η^2^
Time to exhaustion; TTE (s) *						
Pre	481.6 ± 65.5	466.5 ± 87.3	20.0 (2.0–38.1)	5.174	0.031	0.161
Post	484.5 ± 69.8	488.5 ± 98.3				
**HR max**						
Pre	188 ± 11	185 ± 10	1.9 (−1.1–4.8)	1.683	0.206	0.058
Post	189 ± 10	184 ± 11				
**RPE**						
Pre	18.7 ± 1.2	18.7 ± 1.3	0.35 (−0.12–0.81)	2.348	0.137	0.080
Post	18.9 ± 0.9	18.5 ± 1.4				
**Lactate (mmol/L)**						
Pre	7.68 ± 1.98	7.10 ± 1.86	0.22 (−1.43–0.98)	0.144	0.707	0.005
Post	7.42 ± 2.01	7.26 ± 1.99				
**Relative** $\dot{V}$**O_2peak_ (mL/kg/min)**						
Pre	53.2 ± 6.6	52.2 ± 4.8	0.7 (−2.11–0.69)	1.096	0.304	0.039
Post	53.6 ± 6.7	53.4 ± 4.8				
**Absolute** $\dot{V}$ **O_2peak_ (mL)**						
Pre	3956 ± 493	4175 ± 439	0.45 (−112.10–113.01)	2.317	0.140	0.79
Post	3968 ± 467	4265 ± 463				

Data are presented as mean ± SD; *n* = 30 (15 in each group); * denote significant differences between groups.

**Table 5:** Data from the 5 km time trial (TT) before and after the intervention of Haskap or placebo control.

			ANCOVA Adjusted for Baseline			
	Control	Haskap	Difference (95% CI)	F	*p*-Value	Effect Size η^2^
Mean speed (km/h) *						
Pre	13.33 ± 2.06	14.01 ± 1.62	0.25 (0.12–0.38)	15.162	0.001	0.387
Post	13.27 ± 2.08	14.21 ± 1.66				
Time (s) *						
Pre	1377 ± 192	1299 ± 141	20.9 (4.2–37.7)	6.662	0.016	0.217
Post	1384 ± 193	1282 ± 140				
**RPE**						
Pre	18.2 ± 0.7	18.3 ± 1.0	0.31 (−0.22–0.84)	1.402	0.248	0.055
Post	18.5 ± 1.0	18.3 ± 1.1				
**Lactate (mmol/L)**						
Pre	4.95 ± 1.57	6.49 ± 1.93	0.12 (−0.88–1.12)	0.062	0.805	0.003
Post	5.48 ± 1.71	6.27 ± 1.37				
**Maximum HR (bpm)**						
Pre	186 ± 10	186 ± 14	0.3 (−2.6–3.3)	0.054	0.818	0.002
Post	186 ± 10	186 ± 13				
**Mean HR (bpm)**						
Pre	177 ± 13	178 ± 13	0.19 (−0.31–3.34)	0.015	0.905	0.001
Post	175 ± 12	176 ± 12				

Data are presented as mean ± SD; *n* = 27 (13 in the placebo control and 14 in the Haskap group); * denote significant differences between groups.

## Data Availability

Data are kept on the University secure server in line with UK law relating to General Data Protection Regulations and the University’s Research Data Management Policy. Requests for data should be sent to the corresponding author.

## Supplementary Materials

The following supporting information can be downloaded at: https://www.mdpi.com/article/10.3390/nu14040780/s1, Table S1: Dietary macro-nutrient content of the placebo and Haskap groups before test 1 and test 2. Dietary content was estimated from self-reported food diaries. Values are presented as means ± SD. There were no difference between visits or between groups. Table S2: Dietary micro-nutrient content of the placebo and Haskap groups before test 1 and test 2. Dietary content was estimated from self-reported food diaries. Values are presented as means ± SD.
