# Supplementary material for: Improved Endurance Running Performance Following Haskap Berry (Lonicera caerulea L.) Ingestion
Source: Nutrients. 2022 Feb 13;14(4):780. doi: 10.3390/nu14040780 (PMC8877138; doi:10.3390/nu14040780)
Supplement: Supplementary file 1 [file nutrients-14-00780-s001.zip › nutrients-1541398-supplementary.pdf]

Supplementary Table S1. Dietary macro-nutrient content of the placebo and Haskap groups before test 1 and test 2. Dietary content was estimated from self-reported food diaries. Values are presented as means ±SD. There were no difference between visits or between groups.

	**Carbohydrate (g)**	**Protein (g)**	**Fat (g)**	**Total Kcal**	**Fruit and vegetable portions**
**Control**					
Pre	254 ±97	89 ±23	79 ±26	2047 ±587	3 ±2
Post	235 ±101	91 ±49	71 ±32	1819 ±682	3 ±2
**Haskap**					
Pre	213 ±65	102 ±54	70 ±28	1873 ±442	3 ±1
Post	226 ±64	94 ±59	71 ±32	1876 ±658	3 ±2

Supplementary Table S2. Dietary micro-nutrient content of the placebo and Haskap groups before test 1 and test 2. Dietary content was estimated from self-reported food diaries. Values are presented as means ±SD.

	**K (mg)**	**Ca (mg)**	**Mg (g)**	**Fe (mg)**	**Zn (mg)**	**Selenium (µg)**	**Thiamin – B1 (mg)**	**Riboflavin – B2 (mg)**	**Vit B6 (mg)**	**Vit B12 (µg)**	**Vit C (mg)**	**Vit D (µg)**	**Vit E (mg)**
**Control**													
Pre	2530 ±759	904 ±279	281 ±83	11 ±5	10 ±5	71 ±41	2.1 ±1.7	1.7 ±0.9	1.9 ±1.0	4.9 ±2.5	95 ±86	8.8 ±13.6	4.6 ±4.7
Post	2490 ±673	859 ±224	257 ±74	12 ±6	8 ±3	58 ±37	1.6 ±1.1	1.5 ±0.9	1.8 ±0.9	4.2 ±3.3	105 ±88	8.1 ±13.8	4.5 ±5.3
**Haskap**													
Pre	2504 ±1114	864 ±408	277 ±144	9 ±6	8 ±5	86 ±82	1.2 ±0.5	1.6 ±1.0	1.5 ±1.1	6.1 ±5.0	92 ±86	4.7 ±8.4	3.2 ±3.5
Post	2472 ±563	745 ±365	242 ±90	8 ±5	7 ±4	67 ±74	1.2 ±0.4	1.4 ±0.8	1.6 ±0.7	5.0 ±5.2	98 ±105	4.1 ±5.5	3.4 ±5.1
